# Interplay between p53 and non-coding RNAs in the regulation of EMT in breast cancer

**DOI:** 10.1038/s41419-020-03327-7

**Published:** 2021-01-04

**Authors:** Sergey Parfenyev, Aastha Singh, Olga Fedorova, Alexandra Daks, Ritu Kulshreshtha, Niсkolai A. Barlev

**Affiliations:** 1 Institute of Cytology RAS, Saint-Petersburg, 194064 Russia; 2 Department of Biochemical Engineering and Biotechnology, Indian Institute of Technology, Delhi, 110016 India; 3 Almazov Federal North-West Medical Research Centre, St-Petersburg, Russia; 4 Moscow Institute of Physics and Technology, Dolgoprudny, 141701 Russia; 5 Orekhovich Institute of Medical Biochemistry, Moscow, 119435 Russia

**Keywords:** Breast cancer, Long non-coding RNAs, miRNAs

## Abstract

The epithelial–mesenchymal transition (EMT) plays a pivotal role in the differentiation of vertebrates and is critically important in tumorigenesis. Using this evolutionarily conserved mechanism, cancer cells become drug-resistant and acquire the ability to escape the cytotoxic effect of anti-cancer drugs. In addition, these cells gain invasive features and increased mobility thereby promoting metastases. In this respect, the process of EMT is critical for dissemination of solid tumors including breast cancer. It has been shown that miRNAs are instrumental for the regulation of EMT, where they play both positive and negative roles often as a part of a feed-back loop. Recent studies have highlighted a novel association of p53 and EMT where the mutation status of p53 is critically important for the outcome of this process. Interestingly, p53 has been shown to mediate its effects *via* the miRNA-dependent mechanism that targets master-regulators of EMT, such as Zeb1/2, Snail, Slug, and Twist1. This regulation often involves interactions of miRNAs with lncRNAs. In this review, we present a detailed overview of miRNA/lncRNA-dependent mechanisms that control interplay between p53 and master-regulators of EMT and their importance for breast cancer.

## Facts

- Epithelial-to-mesenchymal transition (EMT) is a highly orchestrated transcriptional program that takes place during the development and is mediated by several EMT-specific transcription factors (TFs): Zeb1/Zeb2, Snail, Slug, and Twist1.
- EMT is frequently re-activated during tumorigenesis, including breast cancer, and is responsible for drug resistance and metastasis.
- p53 hinders EMT by augmenting the expression of miRNA-200 that in turn, attenuates Zeb1/Zeb2 levels. The opposite is also true, thus forming a regulatory feedback loop.

## Open questions

- How does mutant p53 affect EMT in breast cancer?
- What are the relationships between p53, long non-coding RNAs, and miRNAs?
- Do p53-dependent miRNAs that target EMT:-TFs identified in various tumors also operate in breast cancer?

## Introduction

### Breast cancer

Breast cancer is the world’s leading cause of cancer-related death in women, impacting >2.0 million women each year worldwide. It is characterized by a high degree of heterogeneity on the clinical, morphological, and molecular levels. Despite its heterogeneity, breast cancer can be broadly categorized into four subtypes: luminal A (ER+, and/or PR+, HER2−), luminal B (ER+, PR+, HER2+), HER2 enriched (ER–, PR–, HER2+), and triple-negative breast cancers (TNBC) comprising basal-like (ER–, PR–, HER2–) and claudin-low breast cancers. The TNBC group is considered to be the most aggressive and highly metastatic type of breast cancer. Despite high incidences, the 5-year survival rate of women with breast cancer is comparatively favorable estimated at ~80–90%, but it rapidly decreases to around 24% in cases where distant metastases are detected. It is also predicted that 30% of early-stage breast cancer patients will progress to the severe forms of the disease, developing remote organ metastases^1^.

The aggressive, basal forms of breast cancer that are highly metastatic have undifferentiated, stem cell-like features correlating to a worse prognosis compared to other subtypes. This process of dedifferentiation and acquisition of an invasive phenotype is mediated by a molecular mechanism called epithelial-to-mesenchymal transition (EMT). The EMT process has been strongly correlated with tumor migration, invasion, and metastases. Since metastases are responsible for almost 90% of deaths in breast cancer, it is not surprising that a lot of effort has been made to study the molecular mechanisms governing the control of EMT and invasion.

### Non-coding RNAs in cancer

Non-coding RNAs (ncRNAs) are RNA products transcribed from the non-protein-coding parts of the genome. Although originally believed to be “junk DNA,” the ever-growing list of publications has defined the central role of expressed ncRNAs in development, differentiation, stress, and pathogenesis. There exists a vast variety of ncRNAs, including miRNAs, lncRNAs, piRNAs, and circRNAs, which efficiently modulate gene expression and importantly, regulate expression levels of each other. In cancers, these ncRNAs are reported to function either as oncogenes or tumor-suppressors depending on the cell-type and cellular context.

miRNAs are 18–24-nt long ncRNAs that primarily work by binding to short complementary sequences in 3′UTRs of their target transcripts. This complementary binding usually results in the degradation or sequestration, and translation repression of the respective base-paired mRNAs. However, some evidence suggests that miRNAs can also be positive regulators of translation. By forming complexes with special proteins, such miRNAs on the contrary, have the potential to stabilize or upregulate the translation of their target transcripts in response to specific conditions and cues. Moreover, because of a short seed sequence, a single miRNA has the capacity to target mRNAs of multiple genes either from the same pathway or across diverse pathways, leading to global changes in the expression patterns, which are typically observed in malignancies. To date, there is a grate list of miRNAs, both intracellular and circulating, that were repeatedly demonstrated to be the biomarkers of BC progression, metastasis formation, drug resistance, and outcome prediction, e.g. miR-21, miR-155, or miR-145^2^.

LncRNAs are longer transcripts (>200 nt), and have diverse mechanisms of action. While the majority of lncRNAs are retained in the nucleus to perform tasks like epigenetic modifications, cis or trans regulation of gene expression, and splicing control, a number of lncRNAs are also translocated to the cytoplasm. These cytoplasmic lncRNAs act as competing endogenous RNAs (ceRNAs) and mediate the sponging of miRNAs via miRNA response elements (MRE), thereby eliminating the inhibition on miRNA target genes. They can also regulate translation by complexing with other proteins. The ability of lncRNAs to form secondary and tertiary structures allows them to exert various roles as scaffolds or decoys for their respective target proteins. In addition, lncRNAs can target other RNAs and metabolites, consequently interfering with gene expression. Like miRNAs, there has been a multitude of studies linking the dysregulation of lncRNAs to cancer. Thus, it was repeatedly shown that abnormal expression of numerous lncRNAs among which, for example, MALAT1, HOTAIR, and DANCR, contributes to BC aggressiveness, metastasis formation, and hence BC patients’ prognosis outcome^3^. Therefore, owing to their clinical implications and networks of cross-talk, both miRNAs and lncRNAs are explored as therapeutic and diagnostic options in cancer and subjected to extensive research.

### Epithelial-to-mesenchymal transition (EMT)

The epithelial-to-mesenchymal transition (EMT) is an evolutionary conserved reversible biological process that typically takes place during the development of organisms and results in a switch from epithelial phenotype to the mesenchymal phenotype. Morphologically, EMT is manifested in loss of cell polarity, adhesive, and tight contacts leading to the detachment of cells from the basement membrane. This is paralleled by massive cytoskeletal rearrangements that consequently change the morphology of epithelial cells to fibroblast-like, spindle-shaped cells and render them motile with an increased potential to invade the surrounding tissue and migrate to distant sites motile with an increased potential to invade the surrounding tissue and migrate to distant sites^4^. While EMT is utmost essential during embryo-development (classified as EMT type-1) and wound healing (EMT type-2), it plays an equally important role in cancer progression and dissemination (EMT type-3), consequently determining the prognosis. Mesenchymal cells produced through EMT have been found to be similar to tumor-initiating CSCs with a high expression of CD44, exhibiting the drug-, apoptosis-, and anoikis-resistant phenotypes^5,6^. Further, these cells are able to avoid oncogene-induced senescence and are immunosuppressive in nature^7^.

It is also worth noting that EMT has a large degree of plasticity, i.e. it can be reversed. This process is called mesenchymal-to-epithelial transition (MET). These evidences support the notion that EMT not only helps cancer cells to invade and metastasize but also confers an enhanced endurance and survival, enabling them to establish new tumors at distant sites.

EMT is activated and stabilized in response to a number of paracrine signals driven by stromal cells, such as fibroblasts, myofibroblasts, and mesenchymal stem cells. These signals and growth factors trigger cascades inside the target cells that culminate in the activation of gene expression pathways that initiate EMT. Cancer cells exploit this program to their benefit by modulating a handful of core transcription factors involved in the direct regulation of EMT, conveniently known as the EMT-TFs. These core EMT-TFs, which include Zeb1, Zeb2, Snail, Slug, and Twist1, are pleiotropic in nature and act in various combinations in different cell types to kickstart EMT. In recent times, non-coding RNAs (miRNAs and lncRNAs) have been discovered to act as critical downstream mediators by modulating a large number of target genes and pathways together to either promote or suppress EMT^8^.

It has also been discovered that breast cancer cells of the basal phenotype are more primed transcriptionally to respond quickly to extracellular EMT-inducing signals like TGF-β, thereby becoming more mesenchymal and dedifferentiated as compared to luminal cells which resist this transition^9^. This indicates that the phenotype of the cell is also an important determinant of its response to EMT-inducers and may explain why basal-like breast cancers are so aggressively metastatic with a bleak prognosis.

### The master-regulators of EMT in breast cancer

As mentioned above, the EMT program is largely regulated at the level of gene expression by several transcription factors, including Zeb1 (Zfhx1a, Bzp, Zfhep, dEF1, TCF8, Nil-2, and AREB6)^10^, Zeb2 (Zfh1, CIP1)^11^, Snail (SNAI1), and Slug (SNAI2)^12^, as well as Twist1^13^. Twist1 belongs to the family of Helix-Loop-Helix (HLH) factors that bind specific DNA sequences called E-box (5′-CANNTG-3′) that are located in the regulatory regions of their target genes. In response to specific signaling cues, they repress transcription of the epithelial genes and promote the expression of mesenchymal genes.

The expression of Zeb1 is undetected in luminal breast cell lines but is observed in basal ones with the highest expression in CD44^high^ cancer stem cells (CSC)^9^. Similarly, while Snail is detected in all subtypes, its expression is also the highest in basal-like cells^14^. A study shows that di-acetylated Twist1 is responsible for activating WNT5A and directly contributes to the invasion and tumorigenicity of basal-like breast cells^15^. Likewise, Slug is also known to suppress the expression of ER-alpha by binding to the E-box motif and is responsible for the migration of triple-negative MDA-MB-231 cells^16^. Cells overexpressing Slug also display the basal-like phenotype^17^. These studies reinforce the concept that the EMT pathway is majorly responsible for driving the malignancy of basal-like and TNBC cells, leading to dismal outcomes.

### Association of p53 and EMT

The p53 tumor suppressor, known as the “genome guardian”, is known to play a key role in preventing tumor development^18^. The p53-coding gene *TP53* is mutated in the vast majority of human tumors (in more than 50% of cases). p53 plays the role of a transcription factor that acts in response to various stress signals, causing cell cycle arrest, cell aging, and apoptosis, as well as controlling the metabolism and antioxidant status of cells^19^. Functioning in the development, p53 limits the plasticity of epithelial cells during EMT. For example, p53 can interfere with the delamination of the neural crest, which usually occurs as a result of triggering EMT^20^.

**Fig. 1:**
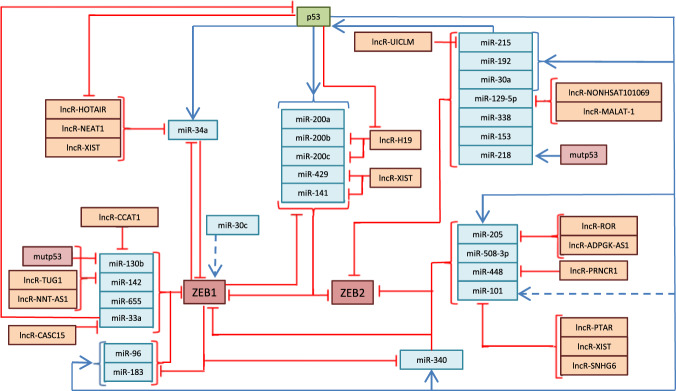
Cross-regulation of p53 and Zeb1/2 EMT-TFs in breast cancer (BC) via miRNAs and lncRNAs. р53 mediates expression of the indicated miRs (denoted with blue color) that in turn target Zeb1/2 EMT-TFs (denoted as burgundy-colored boxes). Long non-coding RNAs (lncRNAs) denoted with pink-color act as competing endogenous RNAs (ceRNAs) to counteract the effect of miRs. Interactions between all of these players form simple links and feedback loops (indicated with arrows and bars). Positive influence is denoted by blue arrows and negative is denoted by red bars.

**Fig. 2:**
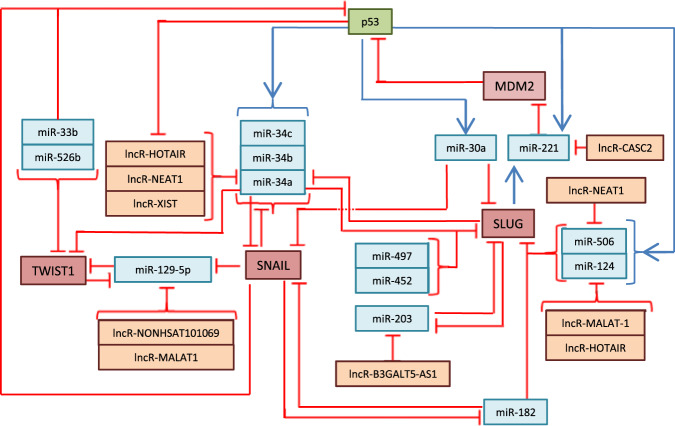
Interactions between p53 and MDM2 in respect to EMT-TFs Twist, Snail, and Slug, which are mediated by miRNAs and lncRNAs in breast cancer. The color-coding is the same as in Fig. 1.

The published studies of the past decade highlighted the novel role of p53 in regulation of metastasis. Wild-type p53 (WTp53) was shown to prevent EMT and the associated stem cell-like phenotype across multiple cancers. As a transcriptional factor, p53 can repress EMT by helping the cells maintain the epithelial gene signature. Furthermore, p53 was shown to induce the attenuation of EMT-TFs levels^21^ via the augmentation of the expression of EMT-suppressing miRNAs. In this respect, it is important to note that p53 induces the expression of miRNAs that target the Zeb, Snail, and Twist families of transcription factors^4^ (Figs. 1, 2). In turn, EMT regulators are shown to attenuate the p53 functions. For example, one of the key EMT inducers Snail) is known to bind to and repress wild-type p53 directly. This Snail-mediated inhibition of p53 was found to be essential for tumor-initiation and growth in breast cancer models^22^.

The importance of p53 in averting cancer metastasis is evident in cases where p53 is lost or worse, mutated. Loss of WTp53 in breast epithelial cells triggers EMT with a parallel increase in the number of cancer stem cells and is associated with a higher tumor grade. This has been attributed to the decrease in expression of tumor-suppressing microRNAs such as miR-200c, which are directly transactivated by p53^23^.

On the other hand, the presence of gain-of-function (GOF) mutant p53 is enough to unleash EMT and makes the cells more migratory in nature. Mutant p53 upregulates the expression of EMT-TFs like Twist1 and Zeb1^24^. This likely occurs because many forms of mutant p53 retain their transcriptional activity albeit with different sequence-specificity^25^. Furthermore, GOF mutants of p53 can mediate its oncogenic effects via the regulation of onco-miRs. This also explains why only the mutant p53 is able to activate multiple oncogenic miRNAs like miR-155 in breast cancer.

Control over the epithelial cell phenotype between p53 and EMT-TFs is a dynamic two-way process. To counteract the repressive effect of p53 on EMT, the EMT-TFs themselves negatively regulate p53 and its regulated miRNAs. For example, Twist1 is known to attenuate the effects of p53 and inhibit cellular apoptosis. Likewise, Snail directly binds and represses p53^22^. As a result of such opposing effects, multiple feedback loops have evolved that include intricate networks of p53, EMT-TFs, and non-coding RNAs. In this review, we attempt to portrait this complex circuitry.

## p53-regulated miRNAs in control of EMT TFs

p53 has been shown to induce the expression of a number of miRNAs that suppress EMT via targeting various EMT-regulating transcription factors, such as Zeb1, Zeb2^4^, and Snail^26^. A list of various miRNAs is shown in Tables 1–3.

**Table 1:** miRNAs targeting Zeb1 and Zeb2.

miRNA	Targets	Cancer subtype	Expression changes	Effect	References	p53 regulation
miR-142-3p	Zeb1	N/S	↓	Downregulated in BC. Reduced expression is associated with tumor size and lymph node metastasis	^98^	Downregulated by mutant p53
				Attenuates breast CSC characteristics	^99^	
miR-96-183 (miR183-96-182 cluster)	Zeb1	N/S	↑	High expression is a prognostic biomarker for BC, and correlated with local relapse, distant metastasis, and poor clinical outcomes	^100^	Induced by p53
		LA	↓	Downregulated in LA BC-induced overexpression of miR-183 inhibited migration of breast cancer cells	^101^	
			↑	miR-96 was upregulated in BC, enhanced proliferation, migration, and tumor growth	^102^	
miR-141 (miR-200 family)	Zeb1 Zeb2	N/S	↑	The level was significantly higher in the blood of patients with stage I–III, lymph node metastasis, and Her2 negative tumors. Low expression of miR-141 was associated with unfavorable overall survival	^103^	Induced by p53
		TN	­	Overexpression promotes migratory and invasive ability of TN BC cells	^104^	
		TN	­	Ectopic expression enhanced brain metastatic colonization	^105^	
		N/S	↑	High level is a marker of circulating tumor cells presence and metastasis	^106^	
miR-200a-200b-200c (miR-200 family)	Zeb1 Zeb2	TN	­	High levels block angiogenesis in tumor	^107^	Induced by p53
		MBC	↑	High levels are associated with metaplastic breast cancer and upregulated Zeb1, Zeb2, Snail, and Slug	^108^	
		N/S	↑	High levels are markers of circulating tumor cells presence and metastasis	^106^	
		TN	↓	Low level is a characteristic of triple-negative BC subtype	^109^	
		LA, LB	↓	Low expression indicated reduced survival in PR-positive BC cases	^110^	
		HER2+, TN	↑	High expression in tumors and increased content in blood were associated with shortened relapse-free survival of patients with in PR-negative BC	^103^	
miR-429 (miR-200 family)	Zeb1 Zeb2	TN	↓	Low level is a marker of BC compared to normal breast tissue. TN breast cancer is characterized by the lowest miR-429 compared to other BC types	^111^	Induced by p53
		TN	-	Upregulated in TN BC (MDA-MB-231 and MDA-MB-468) treated with δ-tocotrienol and induces apoptosis	^112^	
		TN	­	High expression suppresses metastasis formation and migration of MDA-MB-231cells	^113^	
miR-33a	Zeb1	TN, MTC	↓	Downregulated in TN breast cancer	^114^	Suppresses p53 in hematopoetic stem cells
		N/S	–	Expression is negatively associated with lymph node metastasis and clinical stage, expression lower in metastatic cell lines	^65^	
miR-655	Zeb1	TN	↓	Reduced expression in triple-negative BC compared to normal tissue and other cancer types. Lower expression is associated with higher lymph-node status	^115^	
miR-153	Zeb2	LA, TN	­	Ectopic expression inhibited tumor growth and impair the migration and invasion of breast cancer cells	^116^	
		TN	­	Expression was induced by Mifepristone and resulted in suppressed tumor growth of the TN BC cell lines and patient-derived xenografts	^117^	
miR-192	Zeb2	N/S	↓	Decreased in BC tissues	^118^	Induced by p53
		N/S	↓	Decreased in BC tissues and known to inhibit proliferation	^119^	
miR-215	Zeb2	N/S	↓	Downregulated in BC and targets Sox9 lower levels contributed to unfavorable prognosis	^120^	Induced by p53, activates p53
		N/S	↓	Low expression correlated with higher tumor grade, Her2 positive status, lymph node metastasis, and low expression led to lower 5-year DSS (Disease-specific survival)	^121^	
miR-218	Zeb2	N/S	↓	Lower expression of mir-218 is associated with a higher tumor grade, lymph node metastasis and poor prognosis	^122^	
miR-338p	Zeb2	N/S	↓	Downregulated in BC, expression is inversely correlated with lymph node metastasis and TNM stage	^123^	
miR-101	Zeb1, Zeb2	MTC	↓	Downregulated in metastatic BC cells, expression varies amongst different subtypes	^124^	Induced by p53
miR-205	Zeb1, Zeb2	N/S	↓	Downregulated in BC	^125^	Induced by p53
		N/S	↓	Lowest levels in basal-like and invasive TN breast cancers, inhibits CSC-like phenotype by downregulating *ITGA5*	^126^	
miR-340	Zeb1, Zeb2	N/S	­	BC patients with high miR-340 expression unlikely to achieve pathologic complete response	^127^	Stabilizes p53
		TN	­	Inhibits BC progression and metastasis	^128^	
miR-448	Zeb1, Zeb2	N/S	↓	Downregulation is observed in BC patients with chemotherapy-induced EMT	^129^	
miR-508-3p	Zeb1, Zeb2	TN	↓	Expression was decreased in TN BC. Lower miR-508-3p expression significantly associated with lymph node metastasis and distant metastasis	^130^	

*BC* breast cancer, *LA* luminal A subtype, *LB* luminal B subtype, *TN* triple-negative (basal-like) subtype, *HER2+* HER2-positive subtype, *MBC* metaplastic breast cancer, *MTC* metastatic breast carcinomas, *N/S* subtype is not specified, *FBT* familiar breast tumors, *ER* estrogen receptor, *PR* progesterone receptor, *CSC* cancer stem cells.

**Table 2:** miRNAs targeting SNAIL and SLUG.

miRNA	Targets	Cancer subtype	Expression changes	Effect	References	p53 regulation
miR-30a mir-30c	Snail	N/S	↓	Reduced expression is associated with an unfavorable outcome, including late tumor stage, lymph node metastasis, and worse progression	^131,132^	Induced by p53
	Zeb1, Snail, Twist* (*indirectly activates)	FBT TN	↑ ↑	Elevated expression in cancer compared to normal tissue is a characteristic of familiar BC Promotes invasive phenotype	^133,134^	
miR-34a,c mir-34a	Snail	N/S	↑	Associated with positive nodal status, high tumor grade, ER-negativity, and Her2-positivity	^135^	Induced by p53
		TN	↓	Associated with higher tumor grade and reduced patients survival	^136^	
miR-203	Snail	TN	↓	Low level is a characteristic of triple-negative BC subtype	^109^	Induced by p53 in colon and lung cancer
		LA	↓	Low level is a characteristic of luminal A BC subtype	^137^	
		N/S	↑	High level is a marker of presence circulating tumor cells and metastasis	^106^	
miR-124	Slug, CDK4/6	N/S	↓	Downregulated in human breast cancer specimens and the reduced expression is associated with advanced clinical stage and positive lymph node metastasis in breast cancer patients	^138^	Induced by p53
miR-452	Slug	LA	↓	Downregulated in adriamycin-resistant MCF7 cells compared with the parental cell line	^139^	
		TN, LA	↓	Low expression was associated with elevated migration, invasion, and metastasis formation	^140^	
miR-497	Slug	N/S	↓	Low expression was associated with higher differentiation grade, positive Her-2 expression, higher incidence of lymph node metastasis, and advanced clinical stage	^141^	
miR-506	Slug	N/S	↓	Downregulated in malignant breast tissues and its expression is inversely correlated with tumor stage	^142^	Induced by p53
miR-182	Slug, Snail	TN	↑	Upregulated in TN breast cancer tissues, promotes the proliferation, and invasion	^143^	
		TN, HER2+	↑	High expression of circulating mir-182 was the marker of ER- and PR-negative BC	^144^	

*BC* breast cancer, *LA* luminal A subtype, *LB* luminal B subtype, *TN* triple-negative (basal-like) subtype, *HER2+* HER2-positive subtype, *MBC* metaplastic breast cancer, *MTC* metastatic breast carcinomas, *N/S* subtype is not specified, *FBT* familiar breast tumors, *ER* estrogen receptor, *PR* progesterone receptor.

**Table 3:** miRNAs targeting Twist1.

miRNA	Targets	Cancer subtype	Expression changes	Effect	References	p53 regulation
miR-129-5p	Twist1	HER2+	­	High level is associated with sensitivity of BC cells to trastuzumab	^145^	Has potential p53 response elements in promoter
		N/S	↓	Low level is associated with breast cancer diagnosis; Suppresses the proliferation of BC cells	^146^	
		TN	­	Overexpression sensitized MDA-MB-231 cells to irradiation	^147^	
		LA	–	Knockdown of miR-129-5p reduced radiosensitivity of MCF-7 cells		
miR-526b	Twist1	N/S	­	Associated with reduced patients survival and higher tumor grade	^148^	
miR-33b	Twist1	N/S	↓	Downregulated in BC, its expression negatively correlates with lymph node metastasis and tumor stage	^66^	May repress p53 in hematopoetic stem cells

*BC* breast cancer, *LA* luminal A subtype, *TN* triple-negative (basal-like) subtype, *HER2+* HER2-positive subtype, *N/S* subtype is not specified.

### miR 200 family

Kim et al. were the first to show that p53 brings about the suppression of EMT by inhibiting the expression of Zeb1 and Zeb2 through induction of the miR-200 family of miRNAs in hepatocellular carcinoma^4^. Later, Chang and colleagues found that the p53 family directly controls the expression of the miR-200 family in breast cancer^23^.

Several studies have now established that miR-200 family acts a critical mediator of the p53-regulated suppression of EMT by targeting several EMT-TFs including Zeb1 to maintain the epithelial phenotype. All five members of the miR-200 family (miR-200a, miR-200b, miR-200c, miR-141, and miR-429), as well as miR-205, were shown to suppress EMT via targeting Zeb1^27^. The increase in ectopic expression of the genes of this family (miR-200a, miR-200b) leads to morphological changes in cells, which change from a spindle-shaped mesenchymal back to a round epithelial form with concomitant association of cells into groups. In turn, ablation of miR-200 promoted chemoresistance of breast cancer cells during EMT^28^. Loss of the miR-200 family in regions of metaplastic breast cancer specimens was shown to be paralleled with loss of E-cadherin. Thus, downregulation of the members of this miR family is likely to be an important step in the tumor progression.

### Regulation by EMT-TFs: Zeb1 and Snail

Zeb1, along with Snail, plays a critical role in the regulation of p53-regulated miR-200 family members. Zeb1 and Snail have been reported to bind the promoters of miR-200b-200a-429, miR-200c-149, and curb their expression^29^. Additionally, Snail is also involved in promoting the methylation of miR-200 locus, which is essential for sustaining the mesenchymal phenotype in cancer cells.

### Regulation of miR-200 family by lncRNAs

Several p53-suppressed long non-coding RNAs are also involved in the regulation of the miR-200 family, including lncRNA H19 and lncRNA XIST^30,31^. Both lncRNA H19 and lncRNA XIST act as so-called competing endogenous RNAs (ceRNAs), which physically associate with specific miRs through miRNA response elements (MREs) on their target transcripts^32^. Thus, lncRNA H19 and lncRNA XIST inhibit miR-200b and miR-200c^33^, miR-141^30^ and miR-429^31^, respectively.

Overall, the expression of the miR-200 family seems to be under tight regulation by TFs, epigenetic regulators and lncRNAs, and displays a strong effect on the EMT process by targeting the EMT TFs.

### miR-34

It has recently been shown that miR-34a, another target of p53^34^ can directly suppress Zeb1. p53 augments the expression of miR-34a both in cultured cells, as well as in irradiated mice^35^. Notably, Zeb1 can reciprocally inhibit the transcription of miR-34 by binding to the E-boxes located in the promoter regions of miR-34a-b-c^36^. In addition to targeting Zeb1, miR-34a also downregulates Snail, Slug, Twist1, and Notch in metastatic breast cancers^37^, as well as other stemness-associated factors like CD133, CD44, BMI1, and c-Myc^36^ via direct binding 3′UTRs of the respective genes and thereby attenuating the process of EMT and metastasis. Given that miR-34 targets several critical EMT-TFs, it can be considered as a “universal weapon” against EMT.

### Regulation of miR-34 by EMT-TFs: Zeb1, Snail, and Slug

Similar to the miR-200 regulatory mechanism described above, Zeb1 and Snail regulate miR-34a. Siemens and colleagues have established the presence of a negative feedback loop, wherein miR-34a and Snail repress each other by direct binding. This interplay is mediated via the p53-dependent activation of miR-34a during the regulation of EMT^36^. Another member of the Snail family, SLUG has also been reported by De Carolis et al. to directly bind to the promoter of miR-34a and repress its transcription in breast cancer cells exposed to hypoxia, and upregulate the expression of Carbonic Anhydrase isoenzyme 9 (CAI9). This enabled the cells to acquire a stemness like phenotype with an increased ability to form mammospheres^38^.

### Regulation of miR-34 by lncRNAs

It has been noted that several lncRNAs, including lncRNA XIST^39^ and the p53-inducible LncRNA NEAT1^40^ act as ceRNA for miR-34a, which leads to the activation of EMT. The physiological meaning of LncRNA NEAT1 activation by p53, especially in breast cancer, needs to be investigated further.

### miR-192/215 family

Kim et al. demonstrated that the p53-regulated miR-192 was able to prevent EMT in hepatocellular carcinoma by suppressing Zeb2 at the post-transcriptional level^41^. Similarly, in the same study, another p53-dependent miR-215 was shown to significantly reduce levels of mRNA and protein of Zeb2 in cells of the colorectal cancer lines SW620 and DLD-1. Importantly, the observation of EMT-suppressing activity of miR-215 was confirmed in breast cancer cells^42^. However, in this case, the authors focused on Sox-9 as the main target of miR-215. The question of whether miR-215-dependent attenuation of Zeb2 contributed to this process remains to be answered.

### Regulation of miR-215 by lncRNAs

Interestingly, LncRNA-UICLM was shown to compete with miR-215 thereby regulating the expression of Zeb2. lncRNA-CDC6, which is overexpressed in breast cancer tissues, also negatively regulates miR-215 by sponging it. This inhibition of miR-215 promoted the proliferation and migration ability of breast cancer cells^43^.

### miR-30 family

The miR-30 family, which includes five members (miR-30a-e), is involved in the pathogenesis of various types of tumors. It has also been demonstrated that p53 binding to the miR-30a promoter induces the transcription of both miRNA chains—5p and 3p—which are able to interact with Zeb2^44^. Interestingly, the same family members can be either cancer-promoting or tumor-suppressing. For example, miR-30d^45^ and miR-30a have been identified as anti-metastatic factors in different tumors. In an experiment conducted by Mahsa and colleagues using MCF-7 breast cancer cells, an inverse correlation was found between miR-30c and Zeb1. This suggests that miR-30c likely activates Zeb1, although this has not been confirmed by direct experiments^46^. Furthermore, reduced miR-30 expression has been reported to be critical for the maintenance of self-renewal and inhibition of apoptosis in breast tumor-initiating cells.

### miR-124

Mutation in or deletion of the *TP53* gene affects the expression of several miRNAs, among which miR-124 was the most strongly downregulated. This effect correlated with an upregulation of the anti-apoptotic gene, iASPP, suggesting that the latter was the target for miR-124. Consistent with this notion, p53 was shown to bind the promoter of the miR-124 gene to facilitate its expression, which consequently inhibited iASPP expression. Overexpression of miR-124 led to suppression of the CDK4 protein expression and attenuated cell viability, proliferation, and cell cycle progression in MCF-7 and MDA-MB-435S breast cancer cells in vitro^47^.

### miR-124 targets SLUG

The expression of miR-124 is reduced in human breast cancer tissues and its levels have been inversely correlated with the tumor grade. miR-124 attenuated the migration of metastatic breast cancer cell line MDA-MB-231 and reversed the morphology from spindle-shaped to epithelial cobblestone-like, with a parallel increase in the expression of E-cadherin. It was found that miR-124 mediated direct targeting of Slug’s 3′UTR was responsible for the reversal of EMT characteristics^48^.

### Regulation of miR-124 by lncRNAs

LncRNA MALAT-1 serves as a ceRNA for miR-124 in breast cancer^47^ and in the development of non-small cell lung cancer. MALAT1 eliminates the suppressive effect of miR-124 on CDK4 and increases cell proliferation. In addition, lncRNA HOTAIR can also act as ceRNA for miR-124. The authors suggest that HOTAIR activation may enhance the EMT process through inhibition of miR-124^49^.

### miR-203

An important tumor-suppressive miRNA, miR-203, is known to inhibit the invasiveness and migration of breast cancer cells. Two independent studies have shown that miR-203 also targets Slug directly through its 3′UTR^50^. Ding et al. described the presence of a double-negative feedback loop, wherein the promoter of miR-203 itself is targeted and repressed by Slug. In a similar manner, Slug also directly suppresses the expression of members of the miR-200 family^51^. Recently, miR-124 and miR-203 were shown to inhibit the expression of Zeb2 at the post-transcriptional level in human kidney carcinoma cells^52^. It will be interesting to see whether miR-203 can also attenuate the expression of Zeb2 and potentially Zeb1 in metastatic breast cancer cell models.

Studies on keratinocytes have demonstrated the expression of miR-203 to be dependent on p53. In these cells, the knockdown or HPV-mediated degradation of p53 significantly decreased the level of mir-203, while activation of p53 by doxorubicin resulted in the opposite effect^53^. This observation of mir-203’s dependence on p53 was also confirmed in lung cancer cells, wherein the reintroduction of p53 in p53-null cells increased miR-203, while mutant p53 failed to do so. Consequently, the overexpressed miR-203 augmented the sensitivity of both colon and lung cancer cells to gemcitabine-induced apoptosis, possibly by positively regulating the expression of Puma.^54^. Although the association between p53 and miR-203 has not been explored in breast cancer yet, it is very tempting to speculate that such regulation also takes place in BC cells.

### Regulation of miR-203 by lncRNA

LncRNA B3GALT5-AS1, which promoted colon cancer invasion, inhibited miR-203 directly by interacting with the miR-203 promoter^55^. The overexpression of this lncRNA thereby increased the cellular levels of Zeb2 and Slug and promoted EMT.

### miR-129-5p

Tan et al. have reported that the promoter region of miR-129-5p could be potentially targeted by p53, whose malfunction is frequently detected in human cancers^56^. High levels of miR-129-5p increased the expression of E-cadherin by interacting with the 3′UTR region of Zeb2^57^. miR-129-5p levels were significantly decreased in breast cancer cell lines. In a study by Yu et al., Twist1 was shown to be a direct target of miR-129-5p and was repressed on its overexpression. The promoter of miR-129-5p reportedly contains three E-box motifs, and the ChIP analysis confirmed the enrichment of both SNAIL and TWIST1 to these sites, correlating with a decrease in the promoter activity. This suggests the presence of reciprocal negative regulation between Twist1 and miR-129-5p. This downregulation of miR-129-5p via the Twist1-Snail feedback loop stimulates EMT and is associated with poor prognosis in breast cancer^58^.

### Regulation of miR-129 by lncRNAs

The lncRNA NONHSAT101069 was found to function as ceRNA by directly binding to miR-129-5p in breast cancer cells^59^. lncRNA MALAT1 also targets miR-129-5p by directly binding and sponging it, resulting in greater cell invasion and migration. MALAT1 and miR-129-5p were found to have an inverse correlation of expression in TNBC tissues^60^. There have been numerous other reports of MALAT1 regulating multiple modulators and enhancing the EMT and stemness phenotype, which underlines its importance in breast cancer progression^61^. Interestingly, it was found that mutant gain-of-function p53, along with other factors ID4 and SRSF1, associates with MALAT1, and represses the production of an anti-angiogenic splicing isoform of VEGFA, hence promoting angiogenesis in breast cancer^62^.

### The miR-33 family

#### miR-33a-5p targets Zeb1

Surprisingly, miR-33a-5p, which was shown to target p53 in stem cells^63^, also inhibits the expression of Zeb1 by interacting with its 3′UTR region^64^. The overexpression of miR-33a in metastatic breast cancer cells remarkably decreases cell proliferation and invasion in vitro and significantly inhibits tumor growth and lung metastasis in vivo. Conversely, its knockdown in non-metastatic breast cancer cells considerably enhances cell proliferation and invasion in vitro and promotes tumor growth and lung metastasis in vivo, which strongly supports the idea of miR-33 as a tumor suppressor^65^. In hematopoietic stem cells, it is known that miR-33 negatively regulates p53 by binding to two conserved motifs in the 3′UTR of its mRNA. This downregulation of p53 and p53-activated apoptosis is responsible for maintaining the stemness phenotype, and reveals the molecular mechanisms of how miR-33 plays opposing roles depending on the cell type^63^.

#### miR-33b targets Twist1

Twist1 is overexpressed in aggressive breast cancers and is also known to promote breast cancer metastasis to the bone. Lin et al. reported that the levels of miR-33b in human breast cancer tissues are significantly reduced and correlate inversely with node metastasis and tumor stage^66^. MiR-33b was shown to directly bind to the 3′UTR of Twist1 and suppress it. Furthermore, ectopic overexpression of miR-33b in metastatic breast cancer cell lines decreased the number of cancer stem cells (CSCs) and their invasive properties. Their ability to metastasize was also found to be reduced in mice models in vivo, suggesting that this miR plays a vital role in maintaining the stemness and invasion properties of breast cancer cells^66^.

### Regulation of miR33a-5p by lncRNA

Using pull-down analysis with biotin-labeled miR-33a-5p in gastric cancer cells, it was found that miR-33a-5p directly interacted with lncRNA CASC15, establishing its role as a ceRNA for miR-33a-5p^64^. RT-PCR and Western blot analyzes proved that the inhibition of lncRNA CASC15, as well as the induced expression of miR-33a-5p, reduced levels of Zeb1 expression in AGS and SGC7901 gastric cancer cells^64^.

## Other miRNAs

### miR-101

miR-101 is one of the potential targets of p53 in induced pluripotent stem cells^67^. Several studies implicate miR-101 as a regulator of breast cancer, which targets several important oncogenes. By binding to the 3′UTR of the Zeb1 and Zeb2 mRNA sites, it reduces the levels of Zeb1 and Zeb2, which leads to the attenuation of EMT^68^.

The lncRNA PTAR (a pro-transition associated RNA, which is upregulated in mesenchymal subtype cells) has been discovered to inhibit miR-101 activity, acting as ceRNA, and eventually promoting EMT^48^. In addition, lncRNA SNHG6^69^ and lncRNA XIST^70^ also inactivate miR-101 by the same mechanism.

### Regulation of miR-101 by lncRNAs

The lncRNA PTAR (a pro-transition associated RNA) has been discovered to inhibit miR-101 activity, acting as ceRNA and eventually promoting EMT^71^. In addition, another lncRNA SNHG6^69^ and lncRNA XIST^70^ also inactivate miR-101 by the same mechanism.

### miR-205

In breast cancer, p53-inducible miR-205 was shown to possess tumor-suppressive functions^72^. A team of scientists led by Lee^73^ showed that miR-205 inhibits both Zeb1 and Zeb2 mRNAs in the breast cancer cell lines MCF7, MDA231, and SK-BR-3. However, under hypoxic conditions miR-205 in lung cancer promoted EMT by targeting apoptosis-stimulating protein of p53-2 (ASPP2)^74^. This fact indicates that depending on the environment, miR-205 can play either a positive or negative role in the regulation of EMT.

miR-205-5p is under the regulatory control of lncRNAs ROR^75^ and ADPGK-AS1^76^ that inhibit miR-205-5p by acting as ceRNA, promoting cell proliferation, migration, and invasion through stabilizing mRNA of Zeb1. LncRNA-ROR is upregulated in breast tumors, and forced expression of lncRNA-ROR in breast epithelial cells leads to visible changes in morphology, increases mesenchymal markers, activates EMT, and promotes invasion, further generating stem-cell-like cells (CD44^hi^/CD24^lo^) with advanced mammosphere forming ability^75^.

### miR-221

miR-221 is well-known as a basal subtype-specific miRNA and is overexpressed in TNBC cells. It targets the tumor suppressor p27KIP^77^. Noteworthy, in metastatic MDA-MB-231 cells, the miR-221 gene expression was upregulated by Slug, as it was recruited to the E-boxes located in the miR-221 promoter. Conversely, repression of Slug led to decreased levels of miR-221, as well as decreased cell motility^78^.

miR-221 was reported to activate the p53/mdm2 axis by inhibiting Mdm2. In turn, p53 activation was shown to enhance miR-221 expression^79^.

It is important to note that this effect was evident only upon activation of p53 by the cytotoxic drug, doxorubicin. Future studies should elucidate whether the p53-activating effect of miR-221 is still valid in metastatic EMT cells.

In hepatocellular carcinoma, miR-221 (as well as miR-24) is negatively regulated by the sponging action of CASC2, which is a well-established tumor-suppressive lncRNA^80^. It was found that by inhibiting miR-221, CASC2 could mediate the sensitivity of cancer cells to apoptosis induced by anti-cancer agent TRAIL and upregulated Caspase-3, which is a direct target of miR-221.

### miR-506

MiR-506 was shown to target the Slug gene directly and decrease EMT characteristics in breast cancer cell lines^81^. The upstream region of miR-506 has a putative p53 response element. p53 was shown to directly target and upregulate its expression in lung cancer cells^82^, but no such study has been carried out yet for breast cancer.

MiR-506 is also known to induce the demethylation of the MEG3 promoter of a lncRNA MEG3 via SP1/SP2 and Dnmt1. MEG3 is a lncRNA that inhibits cell growth and metastasis of breast cancer cells^83^. The expression of lncRNA MEG3 is reduced in breast cancer tissues and also in cell lines MCF7 and MDA-MB-231. Overexpression of MEG3 also led to a decrease in the Mdm2 RNA and protein levels, which subsequently stabilized p53 on the protein levels resulting in activation of its targets including p21, Maspin, and KAI1^84^. Another lncRNA that plays an important role in regulating miR-506 is lncRNA NEAT1. The gene expression profile data and results of dual-luciferase reporter assay in serous ovarian cancer demonstrated that lncRNA NEAT1 functioned as a competing ceRNA for miR-506 to promote cell proliferation and migration^85^.

### The H19/miR-675 locus

Another long non-coding RNA, LncRNA H19, is highly expressed in metastatic breast cells. The promoter region of LncRNA H19 has been shown to be effectively suppressed by the wild-type p53 protein^86^. Slug upregulates the expression of the H19 locus. Interestingly, this locus also encodes for another miRNA, miR-675. It was found that H19 itself could also upregulate Slug through a mechanism dependent on miR-675. This positive feedback loop increased the invasive properties of cancer cells both in vitro and in vivo^87^.

### miR-10b

MiR-10b is a well-known metastatic miRNA in breast cancer, which is highly expressed in breast cancer tissues and metastatic cell lines. While it was found to have no significant effect on cellular proliferation, the invasiveness of cells, both in vitro and in vivo, significantly increased on miR-10b ectopic expression, and distant metastasis was also promoted. E-box motifs were discovered in the region upstream of miR-10b, and Twist1 was shown to directly bind to one of these regions. Because of this direct binding and their positive correlation of expression, it was suggested that Twist1 drives the expression of miR-10b to promote cancer spread. Downstream, miR-10b targets HOXD10, which is involved in the repression of cell motility genes^88^. However, miR-10b promoter has a p53 response element, and Bisio et al. showed using CHIP assay that p53 was actively recruited to the miR-10b promoter in MCF7 cells and induced its expression^89^. This seems counter-intuitive to the role of miR-10b as a “metastamiR”, and functional studies are lacking to further explore the effects of this association.

Hence, the existence of multiple feedback loops between the EMT-TFs and p53-dependent miRNAs may be responsible for driving the final fate of the cell.

## miRNAs regulated by mutant p53 in EMT

Since wild-type and mutant p53 control different networks of genes, it is not surprising that they also affect different spectra of miRNAs involved in EMT. A list of mutant p53 regulated miRNAs is presented below:

### miR-130b

The mutant p53 control different networks of genes, it is not surprising that they also affect different spectra of miRNAs involved in EMT. For example, mutant p53 exerts oncogenic functions and promotes EMT by directly binding to the promoter of miR-130b (a negative regulator of Zeb1) and inhibiting its transcription. Attenuation of mutant p53 in endometrial cancer cells increased miR-130b expression, leading to repression of Zeb1 and blocking the execution of the EMT program^90^. In contrast, Jia et al. have shown that miR-130b by targeting the tumor suppressor protein Pten, enhanced multiple drug resistance, proliferation, and tolerance to apoptosis of breast cancer cells thereby promoting oncogenesis^91^. The nature of this controversy is currently unknown and requires additional investigation.

Interestingly, lncRNA CCAT1, known to interact with p53, also acts as a ceRNA for miR-130b, augmenting the EMT, cell migration, and invasion^92^. By inhibiting the action of miR-130b, CCAT1 upregulates the expression of Zeb1 and Stat3 in ovarian cancer. While CCAT1 has been observed to be oncogenic in TNBC as well, its association with miR-130b has not been examined in breast cancer yet.

### miR-142-3p

An onco-suppressive miR-142-3p inhibits the proliferation, migration, and invasion of breast cancer cells^93^. It is often downregulated upon overexpression of gain-of-function mutant form of p53 due to hypermethylation of its promoter^94^. Using luciferase reporter assay, it was shown that miR-142-3p interacts with the 3′UTR region of Zeb1 mRNA and reduces the levels of Zeb1 transcript and protein. This result suggests that miR-142 likely represses EMT.

Multiple lncRNAs are able to counteract the EMT-suppressing effects of miR-142. In HCC, the RNA product of the taurine upregulated gene 1 (TUG1) reduces the effect of miR-142-3p on Zeb1 by competitively binding to miR-142-3p, acting as ceRNA^95^. Knockdown of lncRNA-TUG1 was consistent with an increase in miR-142-3p levels and limited the invasion of cells. In breast cancer, it was revealed that another lncRNA NNT-AS1, functions as ceRNA specific for miR-142-3p, thereby restoring Zeb1 and blocking EMT^96^. lncRNA NNT-AS1 was also significantly overexpressed in breast cancer and correlated with poor prognosis.

### miR-218

Expression of another miR-218 is specifically upregulated by mutant p53 (R172H) in mesenchymal cells^67^. The R172H mutant displays a GOF activity during the reprogramming of somatic cells into induced pluripotent stem cells and also increases their oncogenic potential. While miR-218 was upregulated in mutant p53 cells and downregulated in wtp53, the exact role of miR-218 in facilitating this reprogramming has not been detailed yet. Unexpectedly, miR-218 was shown to regulate EMT by inhibiting the expression of Zeb2. Liu et al. investigated the role of miR-214 and miR-218 in breast cancer^97^. The authors found that the aberrant expression of miR-214 and miR-218 were negatively associated with Ki 67, and the expression of miR-218 expression was positively associated with progesterone receptor (PR) in breast cancer tissues. Upon overexpression, the cell proliferation and migration in vitro were decreased and cell apoptosis was induced in breast cancer cells. The authors concluded that miR-214 and miR-218 function as tumor suppressors in breast cancer.

miR-218 also caused the inhibition of tumor growth and metastasis in lung cancer. Taken together, these reports may suggest that exerts different functions depending on the cellular context, i.e. in epithelial cells it serves as a tumor suppressor and in the mesenchymal ones upon the induction of pluripotency it may behave as an oncogene.

## Conclusions

MicroRNAs play a decisive role in EMT, either as effector molecules of major transcription factors or as modulators of their expression. Recently, it has become apparent that miRNAs are among the critical regulators of EMT, with the miR-200 family making the main contribution to the process. This in no way undermines the relevance of other players, however, it should be noted that miRNAs play an important role in the regulation of TFs genes, whose products are master-regulators of the EMT. In addition, due to the large number of miRNAs operating in this process, it seems that the regulation is carried out by the additive principle: even a slight dysregulation in the expression levels of several members of one miRNA family would lead to significant amplification of the effect on the level of protein expression for Zeb/Snail/Twist, subsequently affecting the course of EMT. The regulatory complexity of EMT is further exacerbated by the fact that miRNAs themselves are often regulated by other lncRNAs. These feedback loops and networks of p53 and the EMT TFs, miRNA, and lncRNAs involved in the regulation of EMT are shown in Figs. 1 and 2.

Over the past few years, there has been an increase in the number of articles on the regulation of miRNAs using lncRNA. Based on this, it is likely that in the near future, the list of known miRNA regulators involved in EMT will expand significantly. The p53 tumor suppressor protein is a transcription factor by itself. Thus, perhaps it was not surprising to find out that p53 regulates vital cellular processes, including EMT, by regulating multiple miRNAs. Given the fact that p53 is mutated in more than 50% of all human cancers, the question arises as to whether the mutant p53 regulates different cohorts of miRNAs. In fact, it can be hypothesized that the “onco-miRs” regulated by various mutants of p53 may facilitate EMT, in contrast to wild-type p53. Thus, in order to unravel the complex network of EMT regulation one would take into account the status of p53 and other major tumor suppressors as they may all affect the final outcome of EMT and its reverse process called MET. This knowledge should provide means to consciously intervene with the regulation of EMT as part of the anti-cancer therapy.
